# A Scary Complication: Single-center Study on Management and Outcome of Cesarean Scar Pregnancy

**DOI:** 10.1055/s-0041-1728781

**Published:** 2021-05-12

**Authors:** Beate Hüner, Krisztian Lato, Frank Reister, Wolfgang Janni, Miriam Deniz

**Affiliations:** 1 Department of Obstetrics and Gynecology, Ulm University, Ulm, Germany

**Keywords:** scar pregnancy, therapy scar pregnancy, ectopic pregnancies, cesarean section, hysterectomy

## Abstract

A cesarean scar pregnancy (CSP) is a scary and life-threatening complication of cesarean section (CS). Nevertheless, the incidence of CS is constantly growing. The CSP incidence is 0,15% of pregnancies after CS which represents 6,1% of all ectopic pregnancies in women with condition after CS. Therefore, it should be more present in the clinical daily routine. From mild nonspecific symptoms to hypovolemic shock, diagnosis and therapy must be performed quickly. With the progressive growth of the scar pregnancy, a uterine rupture involves the risk of severe bleeding, and an emergency hysterectomy could be necessary. Prolongation of pregnancy has been successful only in a few cases. We report 11 cases from our hospital in the past 10 years. In the discussion, treatment options of this complication with an increasing incidence, which is associated with serious morbidity and mortality, are presented based on the current literature. Treatment options include drug therapy, but also surgical or combined procedures with radiological intervention.

## Introduction

A scary and life-threatening complication of cesarean section (CS) is a cesarean scar pregnancy (CSP). Nevertheless the incidence of CS is continuously growing, 1 with 30,5% in Germany 2 and even 55,8% in Brazil. 3 This increases the risk of short- and long-term complications, such as postpartum bleeding, uterine rupture, abnormal placental invasion (placenta accreta, increta, percreta), infertility, and ectopic pregnancies. 4 A total of 2% of all pregnancies are ectopic, with an upward trend; since the 1970s, the incidence has increased from 0,5% to 2%. 5 A very rare complication as a result of a previous CS is the implantation of an ectopic pregnancy in the area of the scar, an ectopic scar pregnancy. 4 This is a new form of ectopic pregnancies, first described in 1978. 6 The CSP incidence is 0,15% of pregnancies after CS. This represents 6,1% of all ectopic pregnancies in women with condition after CS. 7 The period for the appearance of scar pregnancy after CS can range from 6 months to 12 years. 8 It is unclear whether the suture technique for closing the uterotomy has an influence on the formation of scar pregnancy. 9 The role of the frequency of previous sections is controversially discussed. 7 10 Other risk factors include repeated uterine curettage, Asherman syndrome, myomectomy, and in vitro fertilization (IVF) therapies. 11

Furthermore, the condition after a cesarean scar pregnancy may increase the risk of recurrence of cesarean scar pregnancy, miscarriage, preterm birth or an abnormal invasion of the placenta. 12 Recently, studies hypothesize that a cesarean scar pregnancy could be a precursor of an abnormally invasive placenta. Although this pathology is often detected primarily in the 2 ^nd^ or 3 ^rd^ trimester, the early invasion of the uterine scar by trophoblastic tissue could be detectable during the first trimester scan. 13

Two forms of scar pregnancies are distinguished. The first form is an ectopic pregnancy in the cesarean scar, which grows to the cervicoisthmic space and toward the uterine cavum (endogenous type or Type I). The second form grows toward the bladder and the abdominal cavity and carries a higher risk of rupture (exogenous type or Type II). 14 15

In the exogenous form of scar pregnancy (Type II), the placental tissue abnormally invades the myometrium as an early form of an adherent placenta. If the pregnancy persists, serious complications are likely to occur. Few cases with successful prolongation of pregnancy have been published. 16 17 However, a hysterectomy had to be performed due to a pathologic placenta invasion. 17

Between the 5 ^th^ and 16 ^th^ week of pregnancy, the scar pregnancy becomes noticeable. Symptoms can be variable. A total of 39% of the patients show mild, nonpainful vaginal bleeding, and 16% of the patients indicate moderate to severe abdominal pain. However, up to 37% of the cases can also be found randomly in asymptomatic patients. 7 The most feared complication is uterine rupture with hemorrhagic shock up to the death of the patient. 8

Scar pregnancy is diagnosed first with the help of transvaginal ultrasound, showing an empty uterus cavum and an empty cervical canal with no contact to the gestational sac. This is located in the anterior part of the lower uterine segment with or without a defect of the myometrium between the gestational sac and the bladder. 9 A three-dimensional (3D) ultrasound or magnetic resonance imaging (MRI) can be used for further diagnosis.

The description of the relationship between the gestational sac of the cesarean scar pregnancy, the previous cesarean scar and the thickness of the anterior uterine wall is very important for the prediction of the outcome and for the planning of further therapies. 18 A cesarean scar pregnancy on top of a well-healed scar had a better outcome than in cases in which the pregnancy was located in the niche of a dehiscent scar. 19

So far, there is no guideline or gold standard for the treatment of scar pregnancies. The discussion compares the various therapies.

## Cases at the Ulm University Hospital between 2009 and 2019

**Fig. 1:**
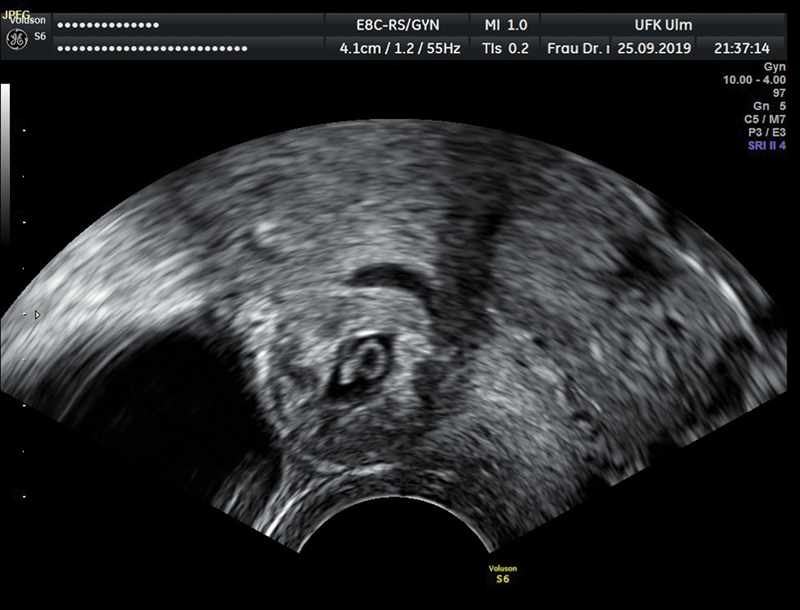
Ultrasound picture of a scar pregnancy.

In the past 10 years, we have treated 11 cases of scar pregnancy due to a previous CS ( Table 1 ). A total of seven patients had only one CS before, one patient was in condition after two CS, and two patients had condition after three sections. The diagnosis was always made with transvaginal ultrasound in the 1 ^st^ trimester when the pregnancy seat was unclear ( Fig. 1 ). In seven cases, a positive heart action was shown. The beta human chorionic gonadotropin (β-HCG) value ranged from 8.000 to 128.000 IU/l. Symptoms ranged from under-period vaginal bleeding to severe lower abdominal pain with over-period bleeding ( Table 2 ).

**Table 1:** Cases

ID	Age (years old)	Gravida	Para	Number of Cesarean Sections	β-HCG value
Case 1	38	4	3	2	16.369
Case 2	37	3	2	1	8.341
Case 3	39	4	4	1	45.651
Case 4	42	3	3	1	73.040
Case 5	30	4	3	3	90.231
Case 6	37	8	3	2	6.303
Case 7	24	2	1	1	57.833
Case 8	29	2	1	1	75.000
Case 9	40	2	1	1	20.365
Case 10	37	3	1	1	128.758
Case 11	28	4	3	3	85.142

**Table 2:** Complications, therapy, and outcome

ID	Bleeding	First-line therapy	Week of pregnancy	Positive heartbeat	Hysterectomy	Additional therapy
Case 1	Yes	MTX 1x IM	9	No	No	Foley catheter, curettage
Case 2	Yes	MTX 1x IM	7	No	Yes	No
Case 3	No	No	6	Yes	No	Laparoscopy (ectopic pregnancy expected in the fallopian tube)
Case 4	Yes	MTX i IM and intralesional	8	Yes	Yes	No
Case 5	No	MTX IM and intralesional	10	Yes	Yes	Mifegyne, cytotec
Case 6	Yes	Hysterectomy	8	Yes	Yes	No
Case 7	No	MTX IV	9	No	No	No
Case 8	Yes	MTX IM	9	Yes	No	Excision with reconstruction of the uterus
Case 9	No	MTX IM and intralesional	7	No	No	Curettage
Case 10	Yes	MTX IM and intralesional	9	Yes	Yes	No
Case 11	No	MTX IM and intralesional	7	Yes	Yes	No

Abbreviations: IM, intramuscular; IV, intravenous; MTX, methotrexate.

In all cases, an individualized therapy planning was discussed; most of the time, initially, with methotrexate (MTX). So far, there is no defined treatment algorithm or participation in a randomized trial in our center.

In 10 cases, a detailed consultation with the patients regarding the various treatment options was held, considering the risks and the option of preserving future fertility. In one case (Case 6), a hysterectomy had to be performed after a short consultation due to severe vaginal bleeding. The treatment was performed both on an outpatient and inpatient basis with close-meshed laboratory controls. In one case (Case 3), the scar pregnancy was discovered by chance during laparoscopic surgery, which initially suspected of an ectopic pregnancy in the fallopian tube and could be removed preserving the uterus. No patient wanted a prolongation of the pregnancy.

A total of nine patients were initially treated with methotrexate (MTX). Three patients received the application intramuscularly (IM) (Cases 1, 2, and 8), and in 1 case (Case 7) an intravenous (IV) therapy was performed. In 5 cases (Cases 4, 5, 9, 10, and 11), MTX therapy was administered IM and intralesional controlled by ultrasound. For five patients who received MTX therapy, a hysterectomy was then performed due to an increasing vaginal bleeding and pain. Of these, four patients had received MTX IM. and intralesional. In three cases, uterus preservation was successful. In two cases, an additional curettage followed, once with the insert of a foley catheter for bleeding control after the curettage. In one case (Case 8) the excision of scar pregnancy with preservation of the uterus was achieved after MTX application.

**Fig. 2:**
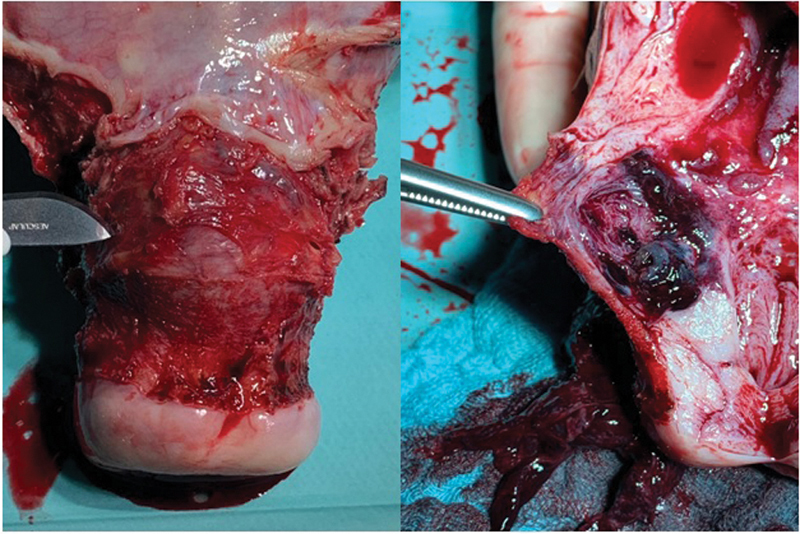
Scar pregnancy removed by laparoscopic hysterectomy.

For one patient (Case 6), due to severe bleeding and pain, no MTX therapy could be performed, and with completed family planning, laparoscopic hysterectomy was performed directly ( Fig. 2 ).

Unfortunately, there is no follow-up to the reproductive outcome after the treatment.

## Discussion

Cesarean scar pregnancy is a very rare form of ectopic pregnancies. As a result, there is no treatment algorithm or gold standard for a therapy. The literature provides mainly case reports or studies with small case series without clear evidence regarding a preferred treatment method. Since 2001, however, the number of published cases has increased in the context of the worldwide rise of CSs. Therapy selection depends on symptoms and associated urgency of the treatment, as well as on the desire for prolongation of the pregnancy, preservation of the uterus, and further family planning. The various options should be discussed in advance with the patient, considering the risks and opportunities of the respective method.

### Expectant Treatment

If desired, an expectant treatment with appropriate clarification can be considered. In particular, the increased risk of uterine rupture and placental implantation disorder with massive blood loss and emergency hysterectomy must be pointed out. In a series of 64 cases, a wait-and-see procedure was performed with 5 patients. In 1 case, there was a live birth in the 36 ^th^ week, discharged by elective CS followed by a hysterectomy due to a placental implantation disorder. 20

A systematic review of 17 studies also examines the wait-and-see strategy. Here, with positive heart action, a high risk of bleeding and, consequently, of surgical intervention in the 1 ^st^ trimester is concluded. A total of 13% of the patients had an uncomplicated abortion, 20% needed surgical intervention, 9.9% suffered a uterine rupture, and 15,2% had a hysterectomy in the 1 ^st^ or 2 ^nd^ trimester. If the pregnancy could be prolonged until the 3 ^rd^ trimester, 3 out of 4 patients presented with placenta percreta. 4

A study of 60 scar pregnancies describes a wait-and-see procedure with intact pregnancies in 10 cases. 17 In 4 cases, there was a live birth by elective CS between the 32 ^nd^ and 36 ^th^ week. In three cases, a hysterectomy had to be performed on placenta percreta. In our collective, no patient wanted a wait-and-see approach.

If a treatment becomes necessary, it should be applied in the 1 ^st^ trimester, as a pregnancy increases the risk of a rupture with massive bleeding and hypovolemic shock. The treatment must be tailored to the maintenance of fertility, gestational age, clinical symptoms, and defects in the area of the myometrium. 9 Treatment options include pharmacological, radiological, surgical, and combined procedures, with up to 30 different options.

### Foley Catheter

A simple method is a curettage with a previous insertion of a foley catheter. In a study on this procedure, 311 patients with an asymptomatic endogenous scar pregnancy up to the 8 ^th^ week were included. A foley catheter was used for compression over 24 hours and the patients underwent a curettage afterwards. Subsequently, β-HCG follow-up checks confirmed the success. The success rate was of 90,7%, with a shorter duration of treatment than with MTX therapy alone. 21 A similar outcome is observed in another study using a double balloon catheter. 22 This method was successfully applied in one of our cases (Case 1) with additional application of MTX IM.

### Methotrexate

The application of MTX is established in the therapy of ectopic pregnancy. This conservative therapy method can also be used in the treatment of ectopic scar pregnancy. Beta human chorionic gonadotropin values < 5.000 IU/I with a dosage of 50mg/m ^2^ show a good response. 23 This treatment is a good option, especially for asymptomatic and hemodynamically stable patients before the 8 ^th^ week without signs of rupture and a myometrium thickness of < 2mm between the scar pregnancy and the bladder. 9

The application can be performed ether IV or IM if necessary, in combination with an intralesional injection. In our collective, MTX therapy was performed in a total of nine patients. In three cases, the therapy was performed IM; in 5 cases, MTX intralesional was applied additionally; and in only 1 case IV therapy was performed. A hysterectomy was ultimately necessary in 5 of the MTX pretreated cases. In four of these cases, a positive heart action was seen in the ultrasound.

### Combined Procedures

In addition to single MTX therapy, the literature also contains a variety of combined methods. For example, in a study with 107 patients, the treatment with curettage alone or with a previous application of MTX IM or IV regardless of the of the β-HCG value. The complication rate did not differ in this study, only longer hospitalization due to MTX treatment was observed. 24

In a larger case that observed 60 patients, the application of MTX intralesional is described for 33 patients in case of positive heartbeat. In addition, the patients received MTX systemically. For bleeding prophylaxis, a foley catheter was inserted after intralesional application. In 31 cases, there was an abortion. With negative cardiac action, no intervention was performed, and 10 patients had a spontaneous abortion. 17 In our sample, we performed an intralesional MTX application in 4 cases with positive heart action. In all cases, a hysterectomy became necessary (see Table 1 , Cases 4, 5, 10, and 11).

The disadvantage of single drug therapy may be the increased risk of bleeding and rupture, as a possibly pre-existing dehiscence of the scar due to the CS or the beginning of rupture of the uterus might not be recognized. 25 If this procedure is planned, it is necessary to know about further family planning.

In a retrospective case control study, the application of MTX in combination with other therapy methods is investigated. A total of 103 patients diagnosed with ectopic scar pregnancy received either MTX in combination with surgical therapy (curettage, laparoscopy, hysteroscopy), MTX in combination with embolization of the uterine artery, or sole surgical intervention. For the application of MTX, a distinction was made between systemic and local administration. Therapeutic effect and outcome were compared. The greatest risk of bleeding and residual surgeries was the sole surgical intervention group. The best treatment option in terms of safety and effectiveness was the combination of local ultrasound-controlled MTX injection and surgical procedures. 26

In another study, 33 patients were treated with either MTX or embolization of the uterine artery or with intraarterial MTX infusion by catheter directly into the uterine artery with subsequent uterine embolization and curettage. Triple therapy is shown here as a more feasible and advantageous variant. 11 This has not yet been performed in our collective. But in the future, with in interdisciplinary cooperation with radiologists, this could be an option for uterus-preserving therapy.

### Operational Procedures

In addition to pharmacological therapy, there is a variety of surgical options for the therapy of scar pregnancies. This includes, among others, laparoscopy with organ-preserving resection, performed for the first time by Lee et al. in 1999. 27 This method is possible with a stable patient and according to surgical experience. In the case of complications from bleeding and hemodynamic instability, a laparotomy must be performed. 9 In our sample, and organ-preserving resection of the scar pregnancy was performed twice (see Table 1 , Cases 3 and 8).

In the literature, there are mainly surgically combined methods. For example, a study examined laparoscopy with hysteroscopy versus curettage with additional embolization of the uterine artery as a basic measure. The sample comprised 58 patients between 2005 and 2010. The group with combination of laparoscopy and hysteroscopy showed a significantly higher resection rate (100 versus 79%), with a lower blood loss (78 ml versus 258 ml), and a satisfactory reconstruction of the cesarean scar pregnancy was possible (96 versus 25%). 28 This can be beneficial in case of further family planning, in order to reduce the risk of rupture in subsequent pregnancies.

In total, > 30 treatment options can be found in the literature. A systematic review highlights five of them. 1 One variant is the removal by transvaginal access based on another case series with 23 patients. This seems to be a promising option for exogenous ectopic pregnancies. 29 Embolization of the uterine artery in combination with curettage and hysteroscopy, as described above as an operationally easy method to perform, can be a low-risk intervention. However, implementation is often limited by the existing infrastructure. A new option seems to be the repeated ultrasound-assisted highly focused ultrasound, but this has not yet been established. Laparoscopy, as a superior method of laparotomy, is particularly suitable for the growth of the pregnancy toward the serosa and for defect restoration of the uterine scar. 1

In summary, an established gold standard is lacking, as well as large multicenter studies. The choice of therapy is therefore mainly dependent on the operational expertise and the availability of the method in the respective clinic.

## Conclusion

Cesarean scar pregnancy is a rare but increasing iatrogenic complication. It should be diagnosed early in the 1 ^st^ trimester. For this purpose, transvaginal ultrasound is suitable. In addition, 3D ultrasound or MRI can be applied. It seems useful to differentiate between endogenous or exogenous scar pregnancy and to consider the thickness of the myometrium to better assess the respective options. Unfortunately, this cannot be reproduced in our collective. In addition, the planning of the treatment depends on existing heart action. Negative cardiac action is more likely to occur in a noncomplicated abortion event. A wait-and-see behavior with positive heart action can only be discussed in individual cases due to serious complications with increasing growth. In the cases described so far, a hysterectomy had to be performed in almost all cases due to an abnormally invasive placenta or to a uterine rupture. The history and risks of the patient should be considered in the preoperative discussion, depending on the family planning and treatment request of the patient. Ideally, the therapy takes place in the 1 ^st^ trimester. Various conservative medications as well as surgical or combined methods, including embolization of the uterine artery, exist. There is no established standard of treatment. The choice of therapy depends on the symptoms, on the location of the ectopic pregnancy (Type I or Type II), on the β-HCG value, and on the desire for fertility preservation. In summary, it can be concluded from our cases that more than half of the women (6 out of 11) had to undergo a secondary hysterectomy, despite drug pretreatment with MTX. Additional intralesional application did not reduce the risk for that. In the presence of a positive heartbeat, a hysterectomy had to be performed in five cases. Combined methods with embolization of the uterine artery have not been used so far, but could represent a new perspective with existing infrastructure. Unfortunately, we do not have a well-established treatment algorithm so far, and an interdisciplinary cooperation is developable. Further large prospective studies with an objective protocol of perinatal diagnostic, management and long term-follow up including the reproductive outcome would be helpful to establish the optimal treatment of cesarean scar pregnancies. Due to the risk of recurrence of scar pregnancy and abnormal invasion of the placenta, an early ultrasound should be performed in specialized centers in further pregnancies. In the future, the risk of this complication after CS will likely increase and should be kept in mind. Early diagnosis, including for asymptomatic patients, as well as transfer to centers with interdisciplinary expertise in the treatment of scar pregnancies is important for the outcome.
